# Editorial: Protein misfolding, altered mechanisms and neurodegeneration

**DOI:** 10.3389/fnmol.2023.1134855

**Published:** 2023-02-01

**Authors:** Neha Gogia, Meghana Tare, Ramakrishnan Kannan, Amit Singh

**Affiliations:** ^1^Department of Genetics, Yale School of Medicine, Yale University, New Haven, CT, United States; ^2^Department of Biological Sciences, Birla Institute of Technology and Science, Pilani, India; ^3^Boyer Centre of Molecular Medicine, Yale School of Medicine, Yale University, New Haven, CT, United States; ^4^Department of Biology, University of Dayton, Dayton, OH, United States; ^5^Premedical Program, University of Dayton, Dayton, OH, United States; ^6^Center for Tissue Regeneration and Engineering at Dayton (TREND), University of Dayton, Dayton, OH, United States; ^7^The Integrative Science and Engineering Center, University of Dayton, Dayton, OH, United States; ^8^Center for Genomic Advocacy (TCGA), Indiana State University, Terre Haute, IN, United States

**Keywords:** Protein misfolding, disease mechanisms, neurodegeneration, human neurodegenerative diseases, proteinopathies, therapeutics, animal models, *Drosophila*

Neurodegenerative diseases (NDs) like Alzheimer's disease (AD), Parkinson's disease (PD), Amyotrophic lateral sclerosis (ALS), Frontotemporal lobe degeneration (FTLD), Polyglutamine diseases such as Huntington's disease (HD), Spinocerebellar ataxias (SCAs) etc., are a group of debilitating disorders that affects millions of people worldwide and have no cure to-date. Despite the advancement in our understanding of molecular and genetic mechanisms underlying these NDs, only a limited symptom-based treatment options are available. As the life expectancy increases there is an increase in the number of ND patients, which will seriously challenge the availability of resources and will impact a nation's economy. There is an urgent need to develop an affordable healthcare system and find effective treatment options to provide better clinical regimens to cure these diseases. NDs affect neurons, neuronal connections associated with memory, cognition, thinking, strength, sensation, movements, learning, co-ordination, and other abilities. Although the causative factors of NDs varies from one to another and the differences in the disease symptoms could be many, these diseases share some common features. One of the common pathological hallmarks among the most NDs is aggregation or deposition of misfolded proteins. Compelling evidence from neuropathological, genetic, animal models studies, and other approaches have strongly supported the fact that accumulation of misfolded protein aggregates triggers a series of detrimental events, which results in synaptic alterations, neuronal cell loss, and significantly contributes toward disease pathogenesis.

This Research Topic highlights the new approaches employed to develop therapeutics, which can effectively block or slow down the onset or progression of these fatal NDs. This manuscripts collection highlights the current advances in the field of neurodegenerative disorders, which may help in addressing some of the unanswered questions pertaining to this Research Topic. This collection of manuscripts is divided into three vital categories: (1) Disease mechanisms, (2) Therapeutic perspectives, and (3) Animal model(s). We hope that this topic may help discern the gaps, connect the missing links, improve our current understanding, knowledge related to this topic and open new avenues of research focuses to improve current treatments options against these deadly yet incurable disorders.

## Disease mechanisms

Among the many fatal NDs, Alzheimer's Disease (AD) is the most prevalent neurological disorder that affects around 6.5 million Americans of age group 65 and older today (2022). This number is expected to increase to 13.8 million by the year 2060, unless proper therapeutics to cure, prevent, or slow down the progression of AD are not developed (2022). Current understanding of the field is that AD occurs due to the presence of aggregates as observed in the brain parenchyma of the dementia patients described by Dr. Alois Alzheimer in his pioneering work (Alzheimer et al., 1995). These aggregates are now known as extracellular deposition of Amyloid-beta (Aβ) plaques, neurofibrillary tangles (NFT), intracellular accumulation of hyper-phosphorylated tau (p-tau) proteins (Selkoe, 2004; Small and Cappai, 2006; Yeates et al., 2019). Though the tau biology has been extensively studied in the field of AD and other NDs, such as, FTLD, however, our current understanding about the precise role of tau in the nucleus and especially the mechanism by which the tau modulates the transcription function has remain limited. In this context, a recent investigation utilized human and mouse brains tissue samples to investigate the effect of changes in the transcriptomic and alternative polyadenylation profiles, modulated by wild-type (WT) and mutant P301L tau protein (Montalbano et al.). The study suggested that tau modulates the gene expression of the transcripts associated with chromatin remodeling and splicing complexes (Montalbano et al.). The WT and mutant P301L tau were found to differentially regulate the transcription and alternative polyadenylation (APA) profiles; and P301L mutation was found to affect the transcription mediated by tau protein (Montalbano et al.).

Another age-associated protein misfolding disorder and a common motor neuron disease is the Amyotrophic Lateral Sclerosis (ALS). Dominant missense mutation in superoxide dismutase 1 (SOD1) and aggregation of misfolded mutant SOD1 has been associated with ALS pathogenesis (Deng et al., 1993; Rosen et al., 1993). Previously it has been shown that the turnover of mutant SOD1 protein(s) is more rapid as compared to the wild-type SOD1 (Hoffman et al., 1996). However, the exact effect of aggregation of SOD1 protein on turnover rate of SOD1 protein is yet to be explored fully. A previous study conducted to examine the rate of SOD1 turnover using mouse spinal cord reported no difference with respect to the amount of aggregation and level of disease progression (Farr et al., 2011). However, the rate of turnover in individual cells containing inclusions of aggregates also remained unexplored. Farrawell and Yerbury utilized NSC-34 cells and investigated this aspect. Based on the findings from their study, the group supported the notion that turnover of mutant SOD1 is faster in comparison to the wild-type SOD1 (Farrawell and Yerbury). However, the study also reported that the turnover and synthesis of SOD1 is impaired in the cells containing insoluble SOD1^A4V^ aggregates following impairment of ubiquitin–proteasome system (UPS), and thereby highlighted the role of UPS dysfunction in ALS pathogenesis (Farrawell and Yerbury).

In this Research Topic, a review by Wodrich et al. provides deeper insights into the physiology of unfolded protein response of endoplasmic reticulum (UPR^*ER*^) and mitochondrial UPR (UPR^*mt*^). The review discusses the crucial roles played by UPR^*ER*^ and UPR^*mt*^ signaling and changes in context of aging and neurodegeneration (Wodrich et al.). Furthermore, the review also highlights the therapeutic strategies targeting UPR^*ER*^ and UPR^*mt*^, that may help improve human health conditions (Wodrich et al.).

Another review in this Research Topic by de Mena et al. highlighted the complex interplay between TAR DNA binding protein-43 (TDP-43, a nuclear RNA/DNA-binding protein) and endoplasmic reticulum (ER) stress. TDP-43 proteinopathy is a major pathological hallmark of ALS and FTLD (Arai et al., 2006; Neumann et al., 2006; Kabashi et al., 2008; Sreedharan et al., 2008; Borroni et al., 2009). Though our current understanding on the physiological roles played by TDP-43 has significantly increased, however, the exact molecular mechanism(s) of action that imparts pathogenic nature to TDP-43, has remained unclear. An in-depth review by de Mena et al., focuses on TDP-43 models and carefully investigates the available literature for the data associated with ER stress and TDP-43 pathology. This group highlighted and discussed the role of TDP-43 WT or mutant overexpression, mislocalization in the activation of UPR and provide rigorous insights on the implications of UPR activation in TDP-43 proteinopathies (de Mena et al.). The review points out the underexplored research areas, highlights the key mechanistic and therapeutic questions which are worthy of investigation. Furthermore, the review also discusses the effect of ER stress and UPR activation in TDP-43 aggregation, post-translational modifications and its interest as potential therapeutic target (de Mena et al.).

## Therapeutic perspectives

A general notion in the field of AD is that the insoluble protein fibrils contribute toward AD etiology and pathophysiology. However, some studies have also suggested that the soluble oligomers or protofibrils can also be toxic, which makes them a target for future disease modifying therapies that can effectively treat AD (Usenovic et al., 2015; Siddiqi et al., 2019). Use of molecules that can dissolve these oligomers or protofibrils, insoluble mature fibrils may potentially help in blocking the onset or progression of AD. Taking this into consideration, Kaku et al. utilized a 179 amino acid long, 20-kDa, cytosolic, mammalian protein (highly conserved) known as Fas Apoptosis Inhibitory Molecule (FAIM) (Schneider et al., 1999). In a study conducted earlier by the same group in year 2020, showed that FAIM decreases the aggregation of mutant SOD1 (ALS associated) (Kaku et al., 2020), however, whether this activity of FAIM is limited to ALS or can also be extrapolated to other NDs (especially AD) remained uncertain. Kaku et al. thus investigated the effect of FAIM on pathogenic Aβ oligomers/fibrils (AD associated) using *in vitro* and cell free system. Results from their study showed that FAIM knockout (KO) cells accumulates the insoluble Aβ fibrils/aggregates (Kaku et al.). Their study also reported that recombinant human FAIM solubilizes the amyloid-β fibrils, generate monomers and suppresses the protein fibrillization/aggregation in a cell free system (Kaku et al.). The recombinant human FAIM was observed to prevent Aβ fibrils, as evidenced from Neuro 2A cells (Kaku et al.). Altogether, the study suggested that FAIM (a single and unique metazoan ATP independent protein) prevents aggregation, induces dissociation of aggregated proteins and is an attractive possibility for therapeutic intervention to treat AD or related dementias (Kaku et al.).

Another common neurological disorder, Parkinson's Disease (or PD, age-associated ND) was initially characterized by Dr. James Parkinson in the year 1817 (Parkinson, 1817). Alpha-synuclein (α-synuclein) misfolding and intracellular aggregates formation are well-known to be associated with PD pathology (Spillantini et al., 1997). Accumulation of a-synuclein and loss of dopaminergic neurons in the substantia nigra pars compacta (SNpc) of the brain are the characteristics neuropathological hallmarks of PD (Kholodilov et al., 1999). An efficient drug delivery to the brain with the drug possessing the capacity to effectively cross the blood brain barrier (BBB) is one of the greatest challenges in NDs, including PD. Use of nanoparticles has gained lot of attention of the researchers in this context. However, the exact effects or implications of using the nanoparticles on α-synuclein aggregates, remained unclear. Previous studies have shown that the recombinant soluble α-synuclein can be induced to form aggregates in the presence of nanoparticles in salt solution (Alvarez et al., 2013; Mohammadi and Nikkhah, 2017; Tahaei Gilan et al., 2019). Thus, to investigate whether the nanoparticles would show a similar effect *in vitro*, Jiang et al. utilized the cell culture model of PD. Results from their study showed that nanoparticles could induce the formation of α-synuclein inclusions that is in part could be dependent on the endo-lysosomal impairment and affinity of α-synuclein to the nanoparticles. Overall, the study suggested that caution should be used while using the nanoparticles to treat PD (Jiang et al.).

## Animal model(s)

Besides understanding the disease mechanisms, another arduous challenge for the researchers studying the human NDs is to recapitulate the exact life stage-matched animal models. Several excellent animal models have been developed and extensively utilized by the researchers to study NDs (Singh and Irvine, 2012). Among them, *Drosophila melanogaster* (*a.k.a*. fruit fly), an invertebrate insect model, has proved to be an excellent model to study NDs. This versatility of *Drosophila* model is due to its fully sequenced genome, shorter life cycle, conservation of the basic genetic machinery, less genetic redundancy, presence of orthologs, or homologs of human disease genes etc. *Drosophila* has been used to model NDs like AD (Tare et al., 2011; Cutler et al., 2015; Sarkar et al., 2018; Deshpande et al., 2019, 2021; Yeates et al., 2019; Irwin et al., 2020), PD (Dawson et al., 2010), ALS (Casci and Pandey, 2015; Gogia et al., 2020), HD (Marsh et al., 2003) etc. Keeping in mind the pathological hallmarks of PD and the importance of using relevant animal models to study PD, Ayajuddin et al., developed and utilized an adult life stage-specific rotenone mediated fly model of PD. It is noteworthy that, the vulnerability of PD is exacerbated by aging, genetic basis, or exposure to the environmental toxins such as pesticides: paraquat and rotenone (Tanner et al., 2011). The *Drosophila* model of PD by Ayajuddin et al., exhibited mobility defects, showed inhibition of mitochondrial complex I activity and dysfunction of dopaminergic (DAergic) neurons. The study also reported reduction in the synthesis of rate limiting enzyme: tyrosine hydroxylase (TH), alterations in the levels of dopamine (DA), as well as its metabolites in the brain (Ayajuddin et al.). Overall, this study indicated that the PD-associated phenotypes validate the robustness of their fly model of PD and that this model would offer great help in discovering the life stage-specific genetic targets of small molecules that can offer DAergic neuroprotection (Ayajuddin et al.). This may potentially help in the development of therapeutics that can effectively treat PD in future (Ayajuddin et al.). Additionally, an another study conducted by Rai and Tapadia also utilized fruit fly model to establish the functional link between heat shock cognate (Hsc70), innate immune response (NF-kB) and neurodegeneration under polyQ conditions.

## Conclusion

In conclusion, the research aimed at elucidating the cellular and molecular commonalities among the NDs and targeting them for therapeutics development, holds great potential. Being a common hallmark among the many NDs, it is important to deeply understand the complex biology behind protein misfolding and the cross talk between misfolded proteins, neurodegeneration, and NDs. The articles (including research and reviews) that build up this Research Topic provides deeper insights into the mechanisms that contribute toward NDs. Articles presented in the therapeutics section discusses the new, effective target(s), and compelling strategies for therapeutic intervention that may help in early diagnosis or treatment of diseases. Findings from these studies may potentially be extrapolated from laboratory setting to the translational or clinical research. Lastly, this Research Topic also covers an article pertaining to the use of an interesting *in vivo* (fly) model of PD that recapitulates the pathological features of PD and may offer great hope to the development of future therapeutics. This Research Topic in Frontiers in molecular neuroscience emphasizes the need of detailed understanding of protein aggregation biology required for the development of efficient therapeutics that may either cure or help slow down the onset or progression of NDs and may substantially contribute toward improving the quality life of ND patients.

Altogether, we strongly believe that this collection will help us appreciate and acknowledge the new developments in the field of NDs and how new strategies can help find the cure for these fatal diseases. We hope that the readers enjoy the broad spectrum of topics covered in this collection.

## Author contributions

NG and AS developed the concept and contributed to manuscript writing and editing. MT and RK were involved in reviewing, editing, and providing comments on the manuscript. All authors approved the final version of the manuscript for publication.
